# Conspecifics, not pollen, reduce omnivore prey consumption

**DOI:** 10.1371/journal.pone.0215264

**Published:** 2019-08-22

**Authors:** S. Rinehart, J. D. Long

**Affiliations:** 1 Department of Biology, San Diego State University, San Diego, California, United States of America; 2 Coastal and Marine Institute Laboratory, San Diego State University, San Diego, California, United States of America; 3 Department of Evolution and Ecology, University of California Davis, Davis, California, United States of America; 4 Department of Ecology, Evolution, and Behavior, The Hebrew University of Jerusalem, Jerusalem, Israel; USDA Agricultural Research Service, UNITED STATES

## Abstract

Pollen can decrease (via reduced consumption) or increase (via numerical response) an omnivores consumption of animal prey. Although pollen can increase predation pressure through numerical responses of omnivores, pollen may also suppress predation by increasing omnivore interactions with conspecifics. Despite this potential, studies of the impacts of pollen on predation by omnivores often overlook the effect of these tissues on intraspecific interactions between omnivores. We designed three studies to examine how *Spartina foliosa* pollen and conspecific density impact scale insect prey consumption by ladybeetle (*Naemia seriata*) omnivores. First, we assessed how pollen impacts scale insect consumption by isolated ladybeetles. Second, we measured how pollen influences ladybeetle prey suppression when numerical responses were possible. Third, because initial experiments suggested the consumption rates of individual ladybeetles depended on conspecific density, we compared per capita consumption rates of ladybeetles across ladybeetle density. Pollen did not influence prey consumption by isolated ladybeetles. When numerical responses were possible, pollen did not influence total predation on prey despite increasing ladybeetle density, suggesting that pollen decreased per capita prey consumption by ladybeetles. The discrepancy between these studies is likely a consequence of differences in ladybeetle density—the presence of only two other conspecifics decreased per capita prey consumption by 76%. Our findings suggest that pollen may not alter the population level effects of omnivores on prey when omnivore numerical responses are offset by reductions in per capita predation rate.

## Introduction

Omnivory (i.e. consuming resources from multiple trophic levels) [1] is ubiquitous within several taxa (e.g. birds, mammals, reptiles, insects, and fishes) and influences the structure and function of communities [2–3]. Interactions between omnivores and their plant and animal prey can account for up to 78% of species’ links in food webs [4]. Despite their prevalence, we lack a basic understanding of how pollen and other plant resources (e.g., seeds) affect interactions between omnivores and their prey in natural systems. Some studies suggest that plant resources decrease prey consumption by omnivores [5–6], whereas others suggest the opposite [7]. This discrepancy may be exacerbated by methodological approaches and the spatial scale of the study [8–9]. In fact, many omnivory studies focus on isolated omnivores feeding on a sub-set of possible resources, which only allows omnivore consumption to depend on resource density and the availability of plant resources [8,10]. Such approaches fail to allow important intraspecific interactions (e.g., mating, cannibalism, and competition) and interspecific interactions (e.g., predation and competition), whose occurrence may be altered by plant resources [9, 11–13]. For example, the availability of pollen reduced the magnitude of predation and intraguild predation on western flower thrips (*Frankiniella occidentalis*) by altering the distribution of predators and intraguild predators on plants [9]. Understanding how plant resources and conspecific density affect prey consumption by omnivores may help predict when and where omnivores exert top-down control on prey populations.

Plant resources may suppress omnivore consumption of prey if these resources are equally (or more) palatable than prey or provide important habitat structure [14–16]. For example, when pollen was available, omnivorous phytoseiid mites (*Iphiseius degenerans*) consume fewer prey (larval *Euseius stipulatus*), leading to lower prey mortality [13]. Similarly, omnivorous big-eyed bugs (*Geocoris punctipes*) consume fewer pea aphids (*Acyrthosiphon pisum*) and are less effective at regulating pea aphid populations when high quality plant resources (lima bean pods) are locally available [5].

In contrast, omnivore prey consumption may increase in the presence of plant resources if these resources increase the local abundance of omnivores through a numerical response (i.e. aggregation and enhanced fitness) [5, 15–18] or by lengthening omnivore persistence in habitats with low prey densities [1, 17, 19–21]. For instance, habitat patches containing high densities of lima bean pods had larger populations of omnivorous big-eyed bugs (*G*. punctipes) and big-eyed bugs were less likely to emigrate from patches containing lima bean pods [17].

Elevated conspecific density, due to omnivore numerical responses to plant resources, may increase the frequency of intraspecific interactions (i.e. interactions between conspecifics such as mating, cannibalism, territoriality, competition) [22–23], thereby decreasing the per capita prey consumption by omnivores [24–26]. For example, increased encounters with conspecifics reduced the per capita predation rate of flatworm predators (*Stenostomum virginanum*) on protozoan prey [26]. Similarly, larval tiger salamanders lower their foraging rates when larger conspecifics are present, likely to minimize their risk of being cannibalized [27]. While the effects of plant resources on intraspecific (e.g., cannibalism) and intraguild interactions have been well-studied (see [9, 11–13]), few studies have aimed to understand how changing omnivore densities (associated with numerical responses to plant resources) can indirectly affect prey population dynamics in the field.

Here, we assessed how pollen and conspecific density affect the foraging behavior of an omnivorous salt marsh ladybeetle (*Naemia seriata*) feeding on scale insects (*Haliaspis spartinae*). Ladybeetles in this system commonly consume over 100 scale insects per week (per capita) and can reach densities of 10–16 adult ladybeetles per 0.25m^2^ [18, 28]. We used laboratory mesocosms to assess the impacts of pollen [i.e. cordgrass (*Spartina foliosa*) flowers] on ladybeetle per capita consumption of scale insects. We paired laboratory mesocosms with a field study to assess the impact of cordgrass flowers on ladybeetle and scale insect density under natural conditions, where numerical responses were possible. Finally, to reconcile our laboratory and field studies, we conducted a laboratory no-choice feeding assay to assess how conspecific density impacts ladybeetle per capita scale insect consumption.

## Methods

### Study system

We assessed how pollen and conspecific density influence the ability of the omnivorous ladybeetle, *Naemia seriata* (hereafter ladybeetle), to suppress populations of its insect prey, the armored scale insect *Haliaspis spartinae* (hereafter, scale insects). Scale insects are specialist phloem-feeders on the foundational salt marsh plant, *Spartina foliosa* (hereafter, cordgrass). We used this ladybeetle-scale insect model system for three reasons. First, ladybeetles in this system are facultative omnivores, as access to cordgrass pollen facilitates ladybeetle survival in the absence of other dietary resources [18]. Specifically, adult ladybeetles provided only access to cordgrass pollen survived 1.97-times longer than ladybeetles provided access to no food resources. This suggests that in the absence of prey resources, adult ladybeetles likely consume cordgrass pollen to promote their longevity. Second, adult ladybeetles show resource-dependent aggregation in the field, with ladybeetles tending to preferentially aggregate to habitats containing both scale insects and cordgrass flowers over habitats lacking these resources [18]. Third, adult ladybeetles often aggregate with conspecifics on cordgrass flowers (S. Rinehart and J.D. Long *unpublished data*), suggesting that cordgrass flowers may be a hub of ladybeetle intraspecific interactions (e.g. mating and territoriality). All field work and species collections for this project were conducted under California Fish and Wildlife collection permit number SC 11084 to Jeremy Long at San Diego State University.

### Effect of cordgrass flowers on scale insect consumption by isolated ladybeetles

To test how Flower Access [2 Levels: Flower Access Present (FA+), Flower Access Absent (FA-)] affects consumption of scale insects by individual adult ladybeetles, we conducted a mesocosm experiment at the San Diego State University Coastal and Marine Institute Laboratory (CMIL). On 20-July-2015, we collected 20 sediment plugs (15 x 15 cm; diameter x deep) each containing a single flowering cordgrass stem infested with scale insects from Sweetwater Marsh (South San Diego Bay; 32° 38’ 15.8”N, 117° 06’ 37.5”W). We observed pollen on all cordgrass stems collected at this time. We planted cordgrass stems and field-collected sediment in 2.6 L plant pots with holes for drainage (Elite Nursery Containers; 300 Series). We used toothbrushes to remove all non-scale insect resources (e.g., leafhoppers) from cordgrass leaves and to standardized initial mean total scale insect density to 559 ± 73 insects stem^-1^ (mean ± SE). We collected ladybeetles from two sites, Sweetwater Marsh and San Dieguito Lagoon (32° 58’ 40.4”N, 117° 14’ 32.8”W). We selected these two sites as 1) ladybeetles, scale insects, and cordgrass plants are common at both sites and 2) we could work in these sites without disturbing clapper rail (endangered bird) habitats. We housed collected ladybeetles for at least one week in the laboratory and provided them scale insects and cordgrass pollen ab libitum prior to use in the study.

We placed all potted plants in an outdoor (N = 20), flow-through seawater table. Plants were rearranged randomly each week. We connected our seawater table to a tidal control system that automatically changed tank tidal conditions [between high (plant pots submerged) and low (plant pots not submerged)] at preset intervals creating tidal conditions like those experienced by cordgrass at Sweetwater Marsh at a tidal height of 1.5 m above sea-level. We let potted plants acclimate to tank conditions for two weeks prior to the experiment.

On 03-Aug-2015, we randomly assigned potted cordgrass plants to a Ladybeetle (Present, Absent) and a Flower Access treatment (FA+, FA-). All treatments had scale insects present (n = 5/treatment). In the Ladybeetle Present treatment, we introduced a single adult ladybeetle into each replicate. We replaced ladybeetles every other week, as we experienced a 10% mortality rate [mortality was calculated using all treatments containing live ladybeetles (n = 10 replicates)] each week. In FA- treatments, we placed cordgrass flowers in 16 x 14 cm Glad Fold-Top plastic bags (The Glad Company; Oakland, California). We secured bags to plants with a cable tie. These bags prevented ladybeetle access to the flowers and thus indirectly manipulated their ability to access cordgrass pollen. In FA+ treatments, we did not restrict ladybeetle access to cordgrass flowers and pollen. However, we controlled for the cable tie by attaching a cable tie to all cordgrass stems in FA+ treatments. Preliminary studies showed that enclosing cordgrass flowers in Glad Fold-Top plastic bags had no effect on scale insect populations after four weeks [Scale insect population density- Bag Present: 573.6 ± 77.3 (mean ± SE); Bag Absent: 530.1 ± 88.5]. We prevented insect dispersal among replicates by covering each entire replicate with nylon insect mesh (54 x 50 cm, height x width, mesh size = 1 mm). We maintained this experiment for 6 weeks until 14-Sept-2015.

To assess the effect of Flower Access and Ladybeetles on scale insect density, we monitored adult and juvenile (hereafter "crawler”) scale insect density every two weeks. Adult and crawler scale insects can be distinguished by their mobility and morphology (e.g. unlike crawlers, adults are immobile and produce a white waxy test). We corrected for natural fluctuations in scale insect density by pairing replicates from the Ladybeetle Present and Ladybeetle Absent treatments and using the formula: P_i_ (A_f_/ A_i_)–P_f_ [29]. Here, P_i_ and P_f_ represent the initial and final scale insect density of Ladybeetle Present treatments and A_i_ and A_f_ represent the initial and final scale insect density of Ladybeetle Absent treatments. This correction allowed us to detangle natural variation in scale insect population dynamics from effects of Flower Access. We then compared our corrected adult, crawler, and total scale insect per capita consumption by ladybeetles between Flower Access (FA+, FA-) treatments using a series of two-sample t-tests. All corrected scale insect consumption data were square root transformed. We conducted all statistical analyses in JMP v. 13 (www.jmp.com).

### Effect of cordgrass flowers on ladybeetle aggregation and scale insect consumption

To assess how Flower Access (FA+, FA-) influences ladybeetle aggregation and consumption of scale insects, we conducted a fully factorial study at San Dieguito Lagoon. On 18-Aug-2016, we established 20–0.25m^2^ circular plots (separated by at least 1m) in a monospecific cordgrass stand infested with scale insects. Although ladybeetles may feed on other prey resources (e.g., leafhoppers), they likely constitute only a small portion of ladybeetle diets, as alternative prey resources are rare compared to scale insects. For example, at the experimental site, scale insect density was 16,177 ± 2,174 per 0.25m^2^ (mean ± SE), while leafhopper density was only 25 ± 2.8 per 0.25m^2^ (mean ± SE; S.A. Rinehart *unpublished data*). Ladybeetles have also never been observed consuming leafhoppers under laboratory or field conditions (S. Rinehart and J.D. Long *personal observation*). All plots started with at least four flowering cordgrass stems, a cordgrass stem density of 22 ± 1.1 (mean ± SE), and zero ladybeetle egg clutches. Once a cordgrass stem flowers, the flower remains on the stem until the plant dies. We randomly allocated plots to each treatment (n = 10). In the FA- treatment, we covered all cordgrass flowers with 16 x 14 cm Glad Fold-Top plastic bags (The Glad Company; Oakland, California) and secured bags in place with a cable tie. In the FA+ treatment, we did not inhibit ladybeetle access to cordgrass flowers. However, we controlled for the presence of cable ties in the FA+ treatment by applying cable ties to all stems included in the study. We used plastic bags to inhibit ladybeetle access to cordgrass flowers rather than mesh bags, as plastic also inhibits the transmission of plant volatile cues [30].

To assess how Flower Access influences ladybeetle aggregation, we monitored the density of all ladybeetle life stages (adults, larvae, and egg clutches) in each plot weekly between 08-Aug-2016 and 22-Sept-2016. We determined the density of ladybeetle life stages using two-minute timed searches. During timed searches, we examined all stems in each plot, starting at the soil-air interface and working toward the apical meristem. All ladybeetle life stage densities were log transformed. We tested for effects of Flower Access on the density of each ladybeetle life stage using separate RM-ANOVAs with Flower Access as a fixed factor and week as the repeated measure.

To understand how Flower Access influences ladybeetle suppression of scale insect populations under field conditions, we recorded scale insect density on two focal cordgrass stems (all focal stems had flowers present) in each replicate on two dates (18-Aug-2016 and 22-Sept-2016). We summed the total scale insect density on focal stems in each plot for both timepoints and used this value to calculate the change in scale insect density per plot over the five-week study (n = 10 per flower access treatment). The change in scale insect density was square root transformed. We then compared the change in scale insect density (per two focal stems) between Flower Access (FA+ vs. FA-) treatments using a two-sample t-test.

### Effect of conspecific density on adult ladybeetle per capita scale insect consumption

Because 1) the impact of flower access on per capita consumption of scale insects (no effect in the laboratory, decreased consumption in the field) and 2) ladybeetle intraspecific interactions (absent in the laboratory, present in the field) varied between our studies, we conducted a no-choice feeding assay to examine the influence of adult ladybeetle density on per capita consumption of scale insects. On 10-Nov-2017, we collected adult ladybeetles and flowering, scale-infested cordgrass stems (clipped at the air-soil interface) from San Dieguito Lagoon two hours prior to the study. We standardized the collected cordgrass stems by selecting plants that had flower present, had 4–5 fully-fledged leaves, and were between 30-40cm in height. Collected stems and adult ladybeetles were transported to the CMIL, where we counted the initial total scale insect density per cordgrass stem [initial scale insect density: 254.7 ± 28.3 (mean ± SE)]. Cordgrass stems were then randomly allocated to each Ladybeetle Density treatment: 0, 1, 2, or 3 per stem. We based the upper Ladybeetle Density treatment on survey data showing that adult ladybeetles tend to aggregate in groups of 3 ± 0.6 individuals per cordgrass stem. Sample size was five for all Ladybeetle Density treatments except the 0 treatment, which had three replicates. Our samples sizes for this study were relatively low because we wanted to limit our impacts in salt marsh habitat, as removing cordgrass plants damages critical habitat for several endangered migratory birds. We then placed the clipped end of each cordgrass stem into its own 13 x 13 cm (height x diameter) cylindrical plastic container filled with 700 ml of tap water (to act as a vase) and enclosed the whole cordgrass stem and plastic container in a 54 x 13 cm (length x width) bag made with white nylon insect mesh (6 mm mesh opening). Finally, we introduced zero, one, two, or three adult ladybeetles to each replicate. We accidentally added four adult ladybeetles to one of the three ladybeetle treatments, and thus removed this replicate from our analysis. All replicates were maintained at a mean temperature of 21.1°C with a 12:12 hour light-dark cycle (85.6 ± 5 μmol photons • m-2 • s -1 (PAR); Philips Natural Light 40W). After three days, we removed adult ladybeetles (no ladybeetles were lost or cannibalized during the study) and counted the final total scale insect density on all stems. We then calculated the total scale insects consumed (between all ladybeetles) and the per capita scale insect consumption of adult ladybeetles in all replicates. Because there was no change in scale insect density in zero ladybeetle replicates during the study (one-sample t-test: t_2.00_ = 0.256, p = 0.589), we removed this treatment from further analysis and attributed all reductions in scale insect density to adult ladybeetle predation. Using our 1,2, and 3 ladybeetle treatments, we tested for the effects of adult ladybeetle density (consistent through the study) on total scale insect consumption and the per capita consumption of ladybeetles using linear regressions with ladybeetle density as the independent factor. Total scale insects consumed (between all ladybeetles) and the per capita scale insect consumption were square root transformed prior to analyses. Unlike the other experiments that were run for multiple weeks, we only ran this experiment for three days because we wanted to test whether conspecific density could impact ladybeetle predation on scale insect populations in the presence of cordgrass flowers. Additional research is needed to determine the presence of temporal variation in such impacts.

On the 2^nd^ and 3^rd^ days of the assay (11-Nov-2017 and 12-Nov-2017), we conducted behavioral observations of ladybeetles in all replicates. On each day, we recorded the location (i.e., plant leaf, plant stem, plant flower, or mesh bag) of each ladybeetle in each replicate between the hours of 08:00 and 10:00 am. We then calculated the number of ladybeetles in each replicate that were on any part of the plant (e.g., leaves, stem, or flower) at the time of observation. We tested for effects of ladybeetle density on the number of ladybeetles on any part of the plant using a RM-ANOVA with Ladybeetle Density as a fixed factor and Observation Day as the repeated measure.

## Results

### Effect of cordgrass flowers on scale insect consumption by isolated ladybeetles

**Fig 1:**
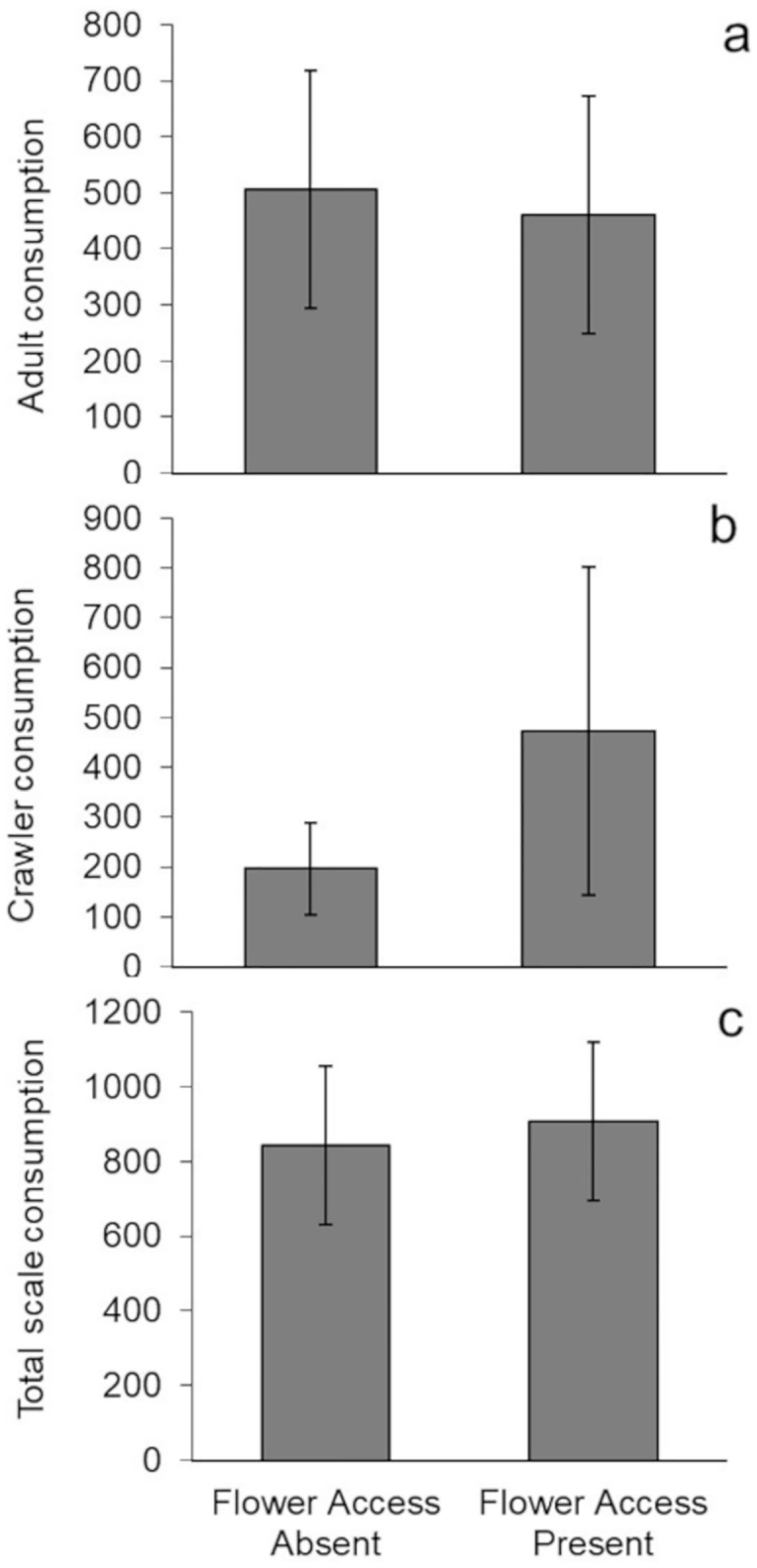
Effect of pollen on isolated ladybeetle foraging. Control corrected mean (± SE) consumption by isolated adult ladybeetles of a) adult, b) crawler, and c) total scale insects (n = 5).

Although ladybeetles have been observed consuming pollen (S.A. Rinehart *personal observation*), consumption of adult, crawler, and total scale insects by isolated ladybeetles was not affected by access to cordgrass flowers (Adults: t_6.59_ = 0.052, p = 0.96; Crawlers: t_5.32_ = 0.596, p = 0.576; Total: t_7.89_ = 0.216, p = 0.834; Fig 1).

### Effect of cordgrass flowers on ladybeetle aggregation and scale insect consumption

**Fig 2:**
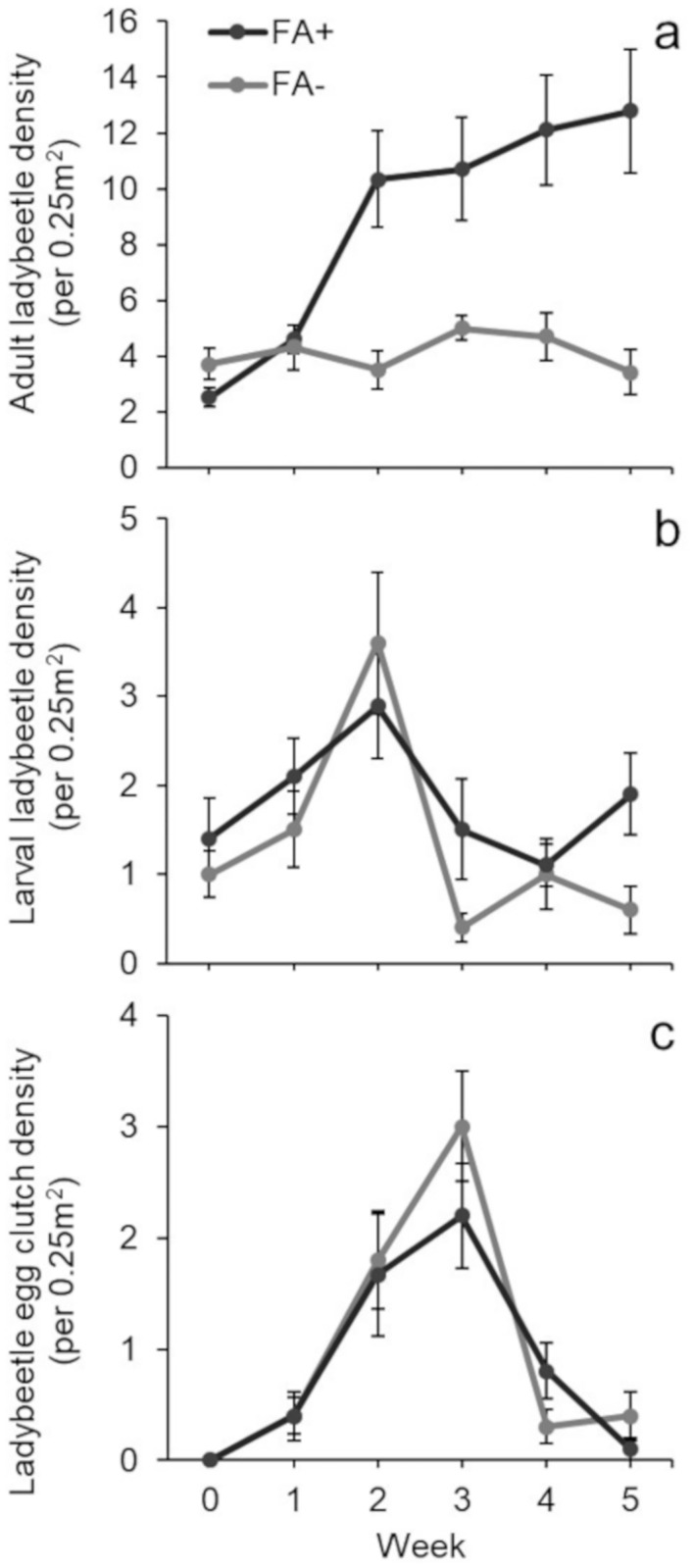
Effect of pollen on ladybeetle population dynamics in the field. Mean (± SE) density of ladybeetle a) adults, b) larvae, and c) egg clutches in 0.25m^2^ manipulated field plots. Flower Access treatments (n = 10) are as follows: Flower Access Present (FA+) and Flower Access Absent (FA-).

In our field experiment, adult ladybeetle density depended on Flower Access (F_1,107_ = 43.69, p <0.001; S1 Table) and week (F_5,107_ = 6.73, p < 0.001). Ladybeetles increased with both factors. Flower Access and week also had an interactive effect on local adult ladybeetle density (F_5,107_ = 6.65, p < 0.001, Fig 2a). This interaction resulted from the differential effects of Flower Access on adult ladybeetle density through time. Specifically, adult ladybeetle density in plots with flower access increased by 412%, while adult ladybeetle density in plots without flower access actually decreased by 8% over the five-week study. Additionally, this effect was strengthened by differences in the initial adult ladybeetle density between treatments, as plots without flower access tended to have more adult ladybeetles than plots with flower access at the start of the study [Initial Adult Ladybeetle Density: FA-: 3.7 ± 0.56 (mean ± SE); FA+: 2.5 ± 0.34 (mean ± SE); Two-Sample T-Test (Factor = Flower Access): t_14.9_ = 1.83, p = 0.087].

Similar to effects on adult ladybeetles, larval ladybeetle density was impacted by Flower Access (F_1,107_ = 5.67, p = 0.019; S2 Table) and week (F_5,107_ = 5.08, p <0.001). Regardless of flower access, larval ladybeetle density peaked in all plots at week two (Fig 2b). However, the presence of cordgrass flowers increased larval ladybeetle density by 36% over the five-week study, while removing access to cordgrass flowers decreased larval ladybeetle density by 40% after five weeks.

The density of ladybeetle egg clutches depended upon time (F_5,107_ = 22.3, p <0.001; S3 Table), with clutch density peaking at week three in both treatments. Flower Access had no effect on egg clutch density (F_1,107_ = 0.38, p = 0.539; Fig 2c), despite adult ladybeetles being 4x more abundant in Flower Access Present plots.

**Fig 3:**
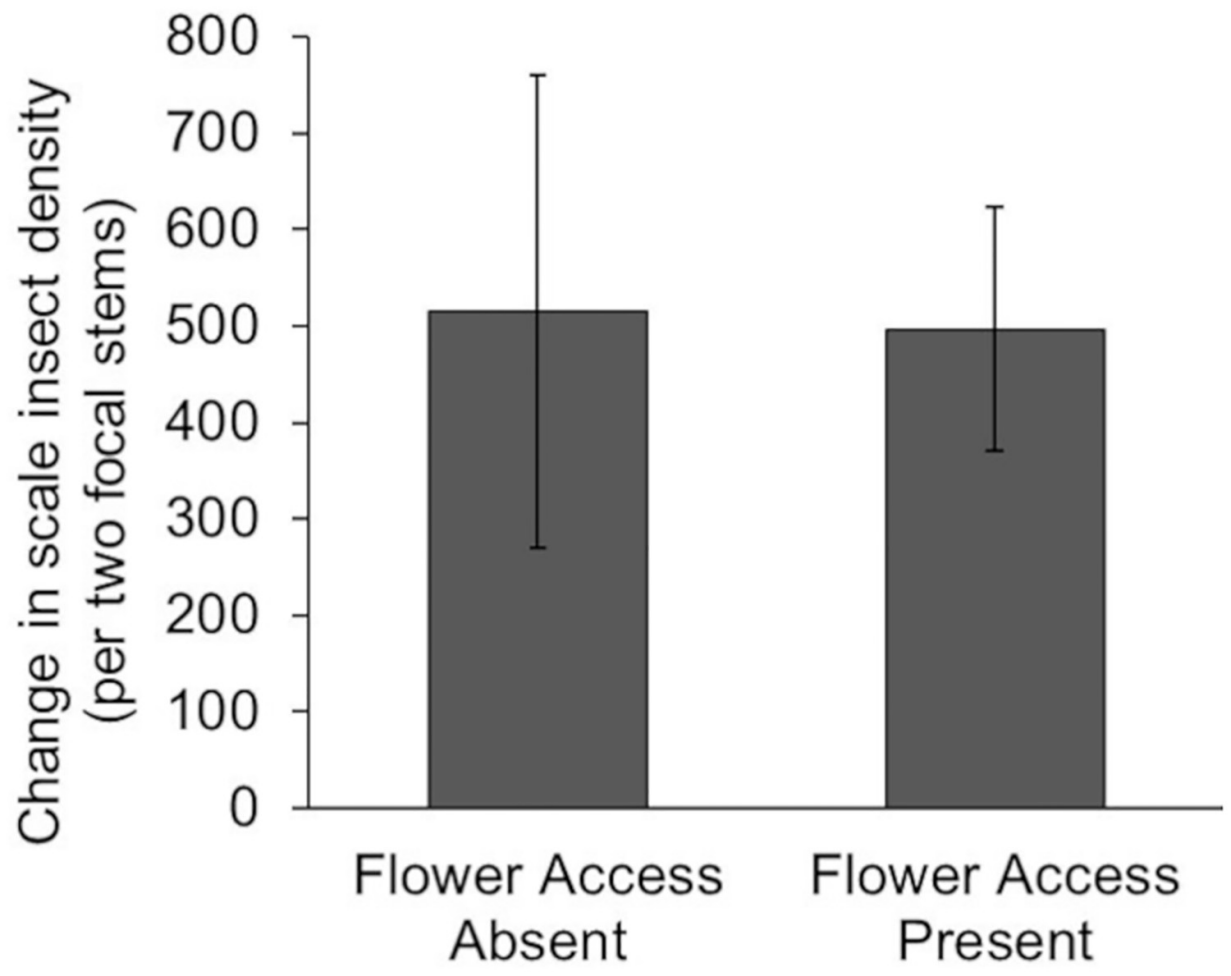
Effect of pollen on ladybeetle foraging in the field. Mean (± SE) change in scale insect density on two focal cordgrass stems in our 0.25m^2^ manipulated field plots (n = 10).

Total scale insect density declined in both treatments over the five-week study (Fig 3). However, there was no difference between Flower Access treatments in the change in scale insect density during the study, despite the higher density of adult ladybeetles in plots with flower access (t_13.07_ = 0.347, p = 0.734).

### Effect of conspecific density on adult ladybeetle per capita scale insect consumption

**Fig 4:**
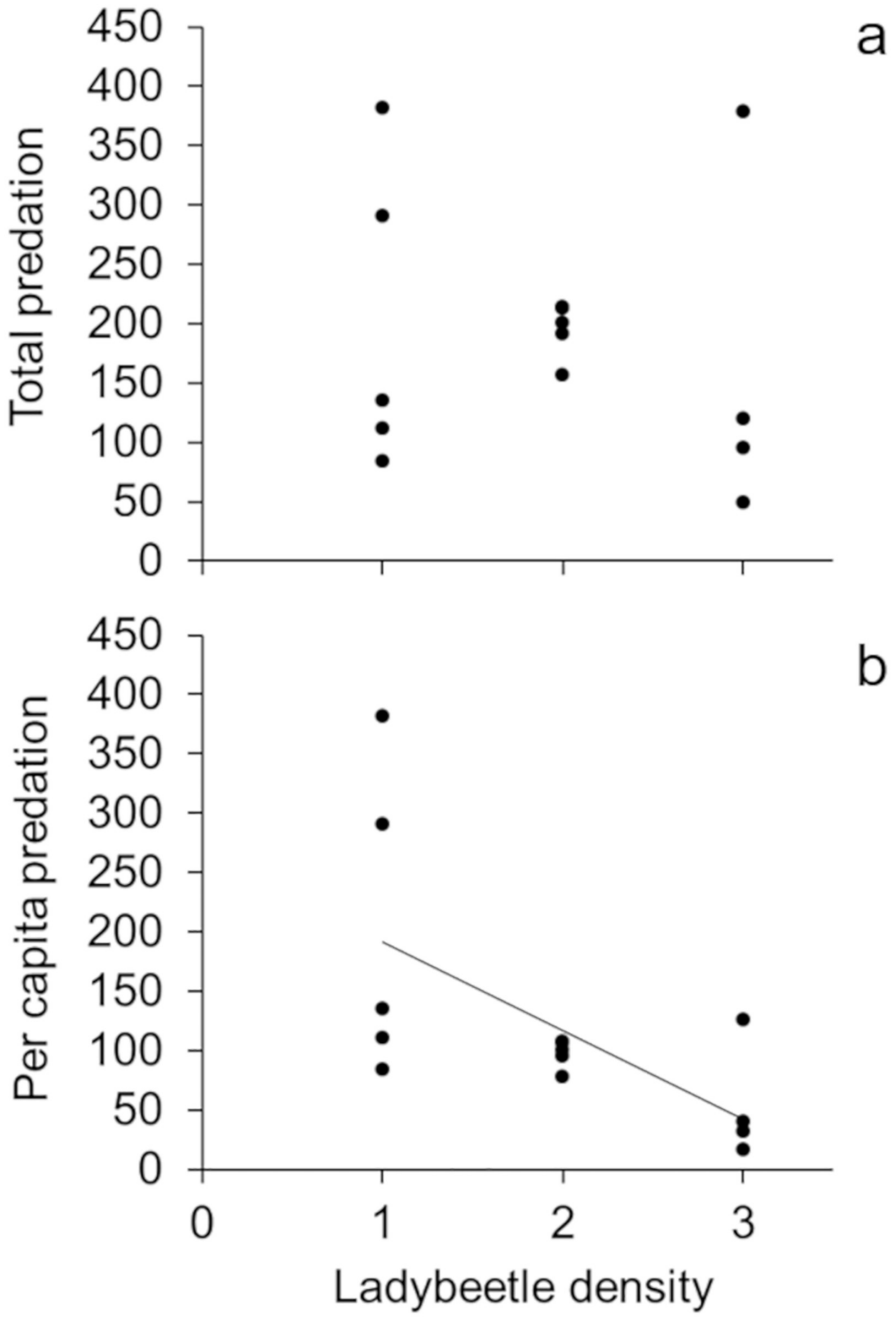
Effect of conspecific density on adult ladybeetle predation rates. Effect of ladybeetle density on **a)** total predation and **b)** per capita predation on scale insects by adult ladybeetles. Sample size five for all ladybeetle density treatments except the 3-ladybeetle treatment (n = 4).

In the presence of 1–3 conspecifics, adult ladybeetle density had no effect on the total number of scale insects consumed (linear regression: *R*^2^ = 0.035, p = 0.520; Fig 4a) and no cannibalistic activities between adult ladybeetles occurred. Additionally, ladybeetles rarely consumed more than 70% of the scale insects provided to them in the trial—suggesting our results were likely due to conspecific density rather than limited prey resources. This suggests that as conspecific density increased, per capita consumption of scale insects declined (linear regression: *R*^2^ = 0.482, p = 0.006; Fig 4b). For example, adult ladybeetle per capita scale insect consumption (over three days) was 201 ± 58 (mean ± SE) for individual adult ladybeetles, but only 48 ± 20 (mean ± SE) for adult ladybeetles with two conspecifics (i.e. three adult ladybeetle treatment).

The number of adult ladybeetles observed on cordgrass plants (i.e., either leaves, stem, or flower) was not affected by Ladybeetle Density (F_2,12_ = 0.68, p = 0.523) or Observation Day (F_1,12_ = 4.00, p = 0.069; S4 Table, S1 Fig). For example, during the first observation day, replicates with 1 ladybeetle had 0.6 ± 0.24 (mean ± SE) adult ladybeetles present on the cordgrass plant, while replicates with 3+ ladybeetles had 1.0 ± 0.32 (mean ± SE) adult ladybeetles on the cordgrass plant. Similarly, on the second observation day replicates with 1 ladybeetle had 0.2 ± 0.2 (mean ± SE) adult ladybeetles present on the cordgrass plant, while replicates with 3+ ladybeetles had 0.4 ± 0.4 (mean ± SE) adult ladybeetles on the cordgrass plant. Thus, regardless of adult ladybeetle density within the replicate, adult ladybeetle density on the cordgrass plant appears to remain constant.

## Discussion

Pollen can increase or decrease prey consumption by altering omnivore foraging behavior. In laboratory mesocosms, isolated adult ladybeetle prey consumption was unaffected by cordgrass flowers (Fig 1). In the field, habitat patches containing access to cordgrass flowers attracted 4x as many adult ladybeetles as habitats lacking flower access (Fig 2a). However, elevated ladybeetle densities in habitats with cordgrass flower access did not result in greater loss of scale insect prey (Fig 3), suggesting that pollen resources reduced ladybeetle per capita consumption of scale insects. This discrepancy (pollen had no effect in the lab but reduced per capita prey consumption in the field), may be related to intraspecific interactions (e.g., interference competition) between ladybeetles which were absent in the lab study with isolated ladybeetles. This hypothesis is supported by our finding that increasing conspecific density reduced per capita consumption of scale insects by ladybeetles. Overall, these observations suggest that pollen may not impact the population level effects of omnivores on prey when numerical responses of omnivores are offset by reductions in their per capita predation rates.

Pollen decreased per capita consumption by omnivores on animal prey in our field study that allowed for intraspecific interactions between omnivores. Although access to cordgrass flowers increased adult and larval omnivore populations (412% and 36%, respectively), this did not translate into a change in animal prey density. Thus, our laboratory study of isolated adult omnivores and our field study contradict each other—access to pollen reduced per capita consumption of animal prey by omnivores in the field but not the lab.

While access to cordgrass flowers increased adult and larval omnivore populations by 412% and 36%, respectively, access to pollen had no effect on the number of omnivore egg clutches. This is surprising, as we expected a greater number of egg clutches in habitats containing larger adult populations. Pollen may have no effect on omnivore egg clutch density for two reasons. First, egg clutches in habitats with pollen may be exposed to increased rates of cannibalism by adult and larval omnivores. Second, omnivores in habitats with pollen may lay more eggs per clutch than omnivores in habitats without pollen; however, this is unlikely in our system as pollen does not increase omnivore per capita egg production [18].

Omnivore predation rates in the presence of pollen may have differed in our laboratory and field experiments for two reasons. First, local environmental conditions may alter omnivore consumption of prey. For example, temperature can directly impact the metabolic rate of ectothermic omnivores, altering their energetic needs and, in turn, their foraging rates. However, we tried to minimize differences in environmental conditions between the laboratory and the field by 1) standardizing the month (August) of both experiments and 2) running our laboratory mesocosm study in outdoor seawater tables with natural tidal cycles (exposing experimental units to the ambient environmental conditions in southern California).

Second, the density of omnivores differed between our first laboratory (single omnivore) and field (multiple omnivores) studies. Differences in omnivore density may explain our conflicting findings if intraspecific interactions (e.g. mating, territoriality, or cannibalism) reduce omnivore consumption of focal prey resources. This seems likely, since our laboratory no-choice feeding assay found that conspecifics reduce ladybeetle per capita scale insect consumption (Fig 4). These findings parallel those of our field study, as ladybeetle populations in cordgrass flower habitats, despite being nearly 4x larger, removed the same number of scale insects as ladybeetles in plots lacking flower access (Figs 2 and 3). A recent meta-analysis suggests that the effects of pollen on omnivore prey consumption depends on the ability of omnivores to elicit numerical responses. Specifically, in the presence of pollen, allowing omnivore numerical responses increased omnivore predation rate on animal prey, while not allowing numerical responses decreased omnivore predation rate on animal prey (Rinehart and Long *in prep*.).

While several studies have aimed to assess the impacts of pollen on intraguild predation and cannibalism (see [9, 11–13]), few have tested how elevated omnivore conspecific density (due to numerical responses to pollen) may affect omnivore foraging behavior and local prey mortality. Here, we found that the presence of only two other conspecifics (i.e. 3+ ladybeetle treatment) decreased per capita prey consumption by 76% in just three days. Omnivores may consume fewer animal prey in the presence of conspecifics if they trade-off between consuming prey and engaging in intraspecific interactions (e.g. mating or interference competition). For example, in our laboratory feeding assay, we frequently observed a single ladybeetle occupying a cordgrass plant at a time- regardless of ladybeetle density (i.e., 1,2, or 3+ ladybeetles). This observation suggests that adult ladybeetles may reduce their predation rates on prey to avoid interacting with other ladybeetles at small spatial scales.

Ladybeetle responses to elevated conspecifics in habitats with pollen resources may also depend on their sex. Increased conspecific density should impact females by increasing intraspecific competition for resources (e.g., prey and pollen) and for oviposition sites; while males should experience increased competition for mates and/or territory [31]. Thus, an important follow-up to this work will be to understand how increased conspecific density impacts the consumptive rates of male and female ladybeetles—as this will provide insight into the mechanisms underlaying the effects of conspecific density on ladybeetle predation rates.

Changes in omnivore conspecific density and the availability of pollen may also influence the rate of cannibalism between ladybeetles in our study system. For instance, post-aggregation cannibalism may explain why adult ladybeetle densities were 4x lower in field plots that denied ladybeetles access to flowers than those allowing ladybeetles access to flowers. However, cannibalism of adult ladybeetles is unlikely in this system for two reasons. First, we never observed cannibalism between individual adult ladybeetles in the laboratory (including during our three-day no-choice assay) or the field (S.A. Rinehart *personal observation*). Second, cannibalistic events are most likely to occur when food resources, especially prey, are limited [12]. In all our studies, ladybeetles never consumed all scale insects in their environment, suggesting that the availability of prey was never limiting.

The effect of pollen on prey consumption by omnivores is commonly attributed to nutritional benefits- as plants and animals vary in their nutrient, vitamin, mineral, and water content [32]. However, pollen may also affect omnivore behavior by increasing habitat complexity. For example, habitat complexity can alter omnivore predation rates and antagonistic intraspecific interactions [33–34]. In our system, ladybeetles preferentially use cordgrass flowers as habitat—field surveys of randomly selected cordgrass stems (n = 95 individual flowering cordgrass stems) found that 88% of adult ladybeetles were found on cordgrass flowers versus other tissues (S5 Table).

The rate of omnivore prey consumption can be influenced by several factors. Historically, omnivory studies have focused on the impacts of pollen on prey consumption and have found evidence that pollen can both increase and decrease the rate of prey consumption by omnivores [5–7]. Pollen can increase local omnivore predation rates by attracting omnivores- as pollen provides omnivores additional food resources and habitat structure [14–16]. However, few studies have tried to understand how local increases in omnivore conspecific density (due to aggregation to pollen) ultimately affect omnivore-prey interactions. Here, we show that omnivore numerical responses to pollen alter the predatory behaviors of omnivores, due to shifts in local conspecific density. Overall, our findings suggest a need to assess the indirect effects of pollen on omnivore predatory behaviors to better understand how omnivory influences food web structure and function.

## Supporting information

S1 Table Repeated measures ANOVA for mean adult ladybeetle density between flower access treatments across the field six-week study. (DOCX)

S2 Table Repeated measures ANOVA for mean larval ladybeetle density between flower access treatments across the six-week field study. (DOCX)

S3 Table Repeated measures ANOVA for mean egg clutch density between flower access treatments across the six-week field study. (DOCX)

S4 Table Repeated measures ANOVA for number of ladybeetles on cordgrass plants between ladybeetle density treatments at two timepoints. (DOCX)

S5 Table Methods and results of ladybeetle habitat use survey, documenting ladybeetle occurrence on flowering and non-flowering cordgrass stems. (DOCX)

S1 Fig Effect of conspecific density on adult ladybeetle behavior in laboratory no-choice feeding assays. Mean (± SE) number of adult ladybeetles observed on cordgrass plant tissues in the laboratory no-choice feeding assay on the 2^nd^ and 3^rd^ days of the assay (n = 5 per beetle density treatment).
(TIF)
