# Supplementary material for: Conspecifics, not pollen, reduce omnivore prey consumption
Source: PLoS One. 2019 Aug 22;14(8):e0215264. doi: 10.1371/journal.pone.0215264 (PMC6705780; doi:10.1371/journal.pone.0215264)
Supplement: S1 Table — (DOCX) [file pone.0215264.s001.docx]

**Supplementary Material:**

**Table S1** Repeated Measures ANOVA for mean adult ladybeetle density between Flower Access treatments across the field six-week study.

| Source of Variation | df | SSQ | F | *P* |
| --- | --- | --- | --- | --- |
| Between subjects |  |  |  |  |
| Flower Access | 1 | 10.17 | 43.69 | **<0.001** |
| Error | 107 | 24.92 |  |  |
|  |  |  |  |  |
| Within subjects |  |  |  |  |
| Week | 5 | 7.84 | 6.73 | **<0.001** |
| Week * Treatment | 5 | 7.75 | 6.65 | **<0.001** |

Adult ladybeetle density was log transformed.
