# Supplementary material for: Conspecifics, not pollen, reduce omnivore prey consumption
Source: PLoS One. 2019 Aug 22;14(8):e0215264. doi: 10.1371/journal.pone.0215264 (PMC6705780; doi:10.1371/journal.pone.0215264)
Supplement: S2 Table — (DOCX) [file pone.0215264.s002.docx]

**Supplementary Material:**

**Table S2** Repeated Measures ANOVA for mean larval ladybeetle density between Flower Access treatments across the six-week field study.

| Source of Variation | df | SSQ | F | *P* |
| --- | --- | --- | --- | --- |
| Between subjects |  |  |  |  |
| Flower Access | 1 | 2.68 | 5.67 | **0.019** |
| Error | 107 | 50.62 |  |  |
|  |  |  |  |  |
| Within subjects |  |  |  |  |
| Week | 5 | 12.02 | 5.08 | **<0.001** |
| Week * Treatment | 5 | 2.46 | 1.04 | 0.400 |

Larval ladybeetle density was square-root transformed.
