# Supplementary material for: Conspecifics, not pollen, reduce omnivore prey consumption
Source: PLoS One. 2019 Aug 22;14(8):e0215264. doi: 10.1371/journal.pone.0215264 (PMC6705780; doi:10.1371/journal.pone.0215264)
Supplement: S3 Table — (DOCX) [file pone.0215264.s003.docx]

**Supplementary Material:**

**Table S3** Repeated Measures ANOVA for mean egg clutch density between Flower Access treatments across the six-week field study.

| Source of Variation | df | SSQ | F | *P* |
| --- | --- | --- | --- | --- |
| Between subjects |  |  |  |  |
| Treatment | 1 | 0.11 | 0.38 | 0.539 |
| Error | 107 | 30.83 |  |  |
|  |  |  |  |  |
| Within subjects |  |  |  |  |
| Week | 5 | 32.12 | 22.30 | **<0.001** |
| Week * Treatment | 5 | 1.43 | 0.99 | 0.425 |

Egg clutch density was square-root transformed.
