# Supplementary material for: Conspecifics, not pollen, reduce omnivore prey consumption
Source: PLoS One. 2019 Aug 22;14(8):e0215264. doi: 10.1371/journal.pone.0215264 (PMC6705780; doi:10.1371/journal.pone.0215264)
Supplement: S4 Table — (DOCX) [file pone.0215264.s004.docx]

**Supplementary Material:**

**Table S4** Repeated Measures ANOVA for number of ladybeetles on cordgrass plants between Ladybeetle Density treatments at two timepoints.

| Source of Variation | df | SSQ | F | *P* |
| --- | --- | --- | --- | --- |
| Between subjects |  |  |  |  |
| Treatment | 2 | 0.87 | 0.68 | 0.523 |
| Error | 12 | 7.60 |  |  |
|  |  |  |  |  |
| Within subjects |  |  |  |  |
| Week | 1 | 1.20 | 4.00 | 0.069 |
| Week * Treatment | 2 | 0.20 | 0.33 | 0.723 |
