# Supplementary material for: Conspecifics, not pollen, reduce omnivore prey consumption
Source: PLoS One. 2019 Aug 22;14(8):e0215264. doi: 10.1371/journal.pone.0215264 (PMC6705780; doi:10.1371/journal.pone.0215264)
Supplement: S5 Table — (DOCX) [file pone.0215264.s005.docx]

**Supplementary Material:**

**Table S5** Number of adult ladybeetles observed on different cordgrass plant tissues on flowering and non-flowering cordgrass stems in San Dieguito Lagoon on Nov. 9^th^, 2017. We sampled cordgrass stems at 0.5m intervals along 6m transects— which were placed perpendicular to the mean low water mark and ran from the lower edge (closest to the water) of the cordgrass patch into the center of the patch. At each 0.5m interval, we selected the cordgrass stem that was closest to the set distance on the transect tape. We then surveyed the cordgrass stem for adult ladybeetles, and recorded their location on the plant (e.g., stem or leaf). Due to it being late in the season— ladybeetle density was relatively low. For example, the adult ladybeetle density per cordgrass stem was 0.42 ± 0.06 in September 2015 (Rinehart and Long 2018), while the density per stem was only 0.13 during this survey.

| Stem type | Total stems  surveyed | Number of adult ladybeetles | | |
| --- | --- | --- | --- | --- |
|  |  | Stem | Leaf | Flower |
| Flowering | 97 | 1 | 1 | 12 |
| Non- flowering | 13 | 0 | 0 | NA |
