# Supplementary figures and images for: Conspecifics, not pollen, reduce omnivore prey consumption
Source: PLoS One. 2019 Aug 22;14(8):e0215264. doi: 10.1371/journal.pone.0215264 (PMC6705780; doi:10.1371/journal.pone.0215264)

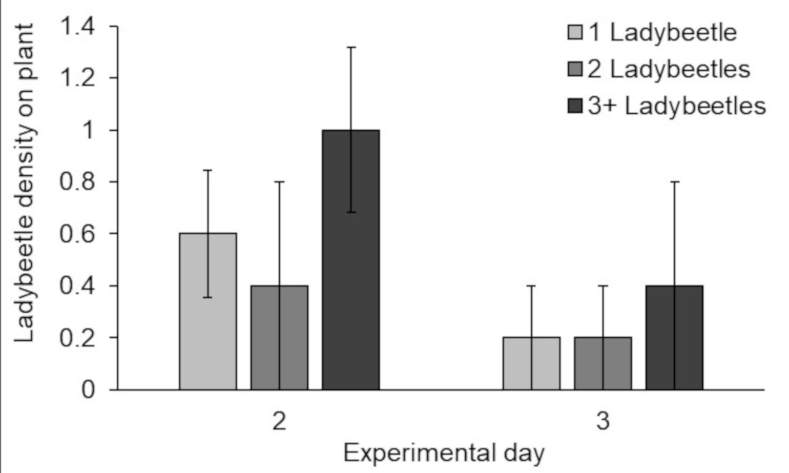

Supplement: S1 Fig — Mean (± SE) number of adult ladybeetles observed on cordgrass plant tissues in the laboratory no-choice feeding assay on the 2nd and 3rd days of the assay (n = 5 per beetle density treatment). (TIF) [file pone.0215264.s006.tif]
